# Virtual reality relaxation for the general population: a systematic review

**DOI:** 10.1007/s00127-021-02110-z

**Published:** 2021-06-13

**Authors:** Simon Riches, Lisa Azevedo, Leanne Bird, Sara Pisani, Lucia Valmaggia

**Affiliations:** 1 Department of Psychology, Institute of Psychiatry, Psychology & Neuroscience, King’s College London, De Crespigny Park, London, SE5 8AF UK; 2 Social, Genetic and Developmental Psychiatry Centre, Institute of Psychiatry, Psychology & Neuroscience, King’s College London, London, SE5 8AF UK; 3 South London and Maudsley NHS Foundation Trust, Bethlem Royal Hospital, Monks Orchard Road, Beckenham, BR3 3BX Kent UK; 4 Department of Psychosis Studies, Institute of Psychiatry, Psychology & Neuroscience, King’s College London, London, SE5 8AF UK; 5 Division of Psychology and Mental Health, Faculty of Biology, Medicine and Health, School of Health Sciences, University of Manchester, Manchester, M13 9PL UK

**Keywords:** Virtual environment, Relaxation technique, Stress management, Restoration, Wellbeing, COVID-19

## Abstract

**Purpose:**

Relaxation has significant restorative properties and implications for public health. However, modern, busy lives leave limiting time for relaxation. Virtual reality (VR) experiences of pleasant and calming virtual environments, accessed with a head-mounted display (HMD), appear to promote relaxation. This study aimed to provide a systematic review of feasibility, acceptability, and effectiveness of studies that use VR to promote relaxation in the general population (PROSPERO 195,804).

**Methods:**

Web of Science, PsycINFO, Embase, and MEDLINE were searched until 29th June 2020. Studies were included in the review if they used HMD technology to present virtual environments that aimed to promote or measure relaxation, or relaxation-related variables. The Effective Public Health Practice Project (EPHPP) quality assessment tool was used to assess methodological quality of studies.

**Results:**

6403 articles were identified through database searching. Nineteen studies published between 2007 and 2020, with 1278 participants, were included in the review. Of these, thirteen were controlled studies. Studies predominantly used natural audio-visual stimuli to promote relaxation. Findings indicate feasibility, acceptability, and short-term effectiveness of VR to increase relaxation and reduce stress. Six studies received an EPHPP rating of ‘strong’, seven were ‘moderate’, and six were ‘weak’.

**Conclusions:**

VR may be a useful tool to promote relaxation in the general population, especially during the COVID-19 pandemic, when stress is increasing worldwide. However, methodological limitations, such as limited randomised controlled trials and longer-term evidence, mean that these conclusions should be drawn with caution. More robust studies are needed to support this promising area of VR relaxation.

**Supplementary Information:**

The online version contains supplementary material available at 10.1007/s00127-021-02110-z.

## Introduction

Relaxation is a state of calmness that helps to release the body and mind from tension [1]. Systematic reviews indicate that relaxation techniques are cost-effective, safe, and practical; they can be easily taught and used as stress management to enhance general wellbeing and mental health [2]. Techniques and practices, such as progressive muscle relaxation, guided imagery, deep breathing, yoga and meditation can be utilised to foster a state of relaxation and positive wellbeing [2], and target a broad spectrum of health and functioning, with evidence for improvements in cognition, respiration, cardiovascular disease, body mass index, blood pressure, diabetes, and joint disorders [3]. With these techniques and practices, elevated heart rate and blood pressure can return to normal levels and psychophysiological arousal can be counteracted with more positive emotions, potentially reducing psychopathological symptoms, psychological distress, and improving subjective wellbeing [1].

Modern life can hinder relaxation practices, with busy schedules and time constraints limiting opportunities and optimal environments for relaxation; while limited time for working aged adults to relax has been shown to exacerbate stress, with evidence of a relationship between elevated stress and an inability to relax [4]. On average, problems related to stress affect one in six working adults [5]. Prolonged exposure to stressors or chronic stress is linked to physical health conditions, such as cardiovascular disease, diabetes, cancer, and autoimmune syndromes [6], as well as psychological distress, depression, anxiety, and substance abuse [7]. Furthermore, COVID-19 is exacerbating stress across the world [8]. Given these obstacles to relaxation, and the huge need to mitigate the psychological impact of stress during the pandemic [9], innovative interventions are needed.

Virtual reality (VR) is at the forefront of technological advancements in mental health care [10]. ‘VR’ typically refers to immersive and interactive head-mounted display (HMD) technology [11], which offers accessible ways to enable relaxation through visualisation, engagement, and immersion with pleasant virtual environments [12, 13]. Experiencing calm virtual audio-visual environments removes users from stressful situations, aiding stress management and relaxation amidst the challenges of everyday life. Increases in relaxation, as well as decreases in stress, arousal and anxiety, have been shown to result from exposure to pleasant virtual environments [13, 14].

Previous systematic reviews have focussed on traditional relaxation techniques targeted at people with health conditions [1, 2, 15]. Despite existing evidence supporting the restorative effects of experiencing pleasant virtual environments, systematic reviews to date have not synthesised studies of HMDs that use virtual environments to support or promote relaxation in healthy participants from the general population [16]. This systematic review aims to synthesise the evidence on the feasibility, acceptability, and effectiveness of HMD relaxation in promoting relaxation in the general population (PROSPERO 195,804).

## Methods

### Search strategy

This review was carried out in accordance with Preferred Reporting Items for Systematic Reviews and Meta-Analyses (PRISMA) [17]. Findings were synthesised using a narrative approach. Web of Science, PsycINFO, Embase, and MEDLINE were searched until 29th June 2020. Search terms were: “virtual real*” OR “virtual-real*” OR “VR” OR “virtual enviro*” OR “virtual character*” OR “VCs” OR “avatar*” AND “relax*” OR “autogen*” OR “meditat*” OR “mindful*” OR “rest*” OR “PMR” OR “progressive muscle” OR “imagery” OR “breath*” OR “distract*” OR “wellness” OR “wellbeing” OR “well-being”. Databases were searched for keyword, title, and abstract information. When searching PsycINFO on the Ovid platform, the ‘explode’ function was used to search key subject headings. Database searches were limited by journal articles and English language. Data were extracted and screened with reference management software Endnote. Dissertations, conference proceedings, and abstracts were excluded. Reference lists of key papers were screened. Two reviewers (LA, SP) independently conducted all searching and screening in consultation with other researchers (SR, LB).

### Inclusion and exclusion criteria

Studies were included in the review if they (a) were published in a peer-reviewed journal; (b) were written in English; (c) included an experimental study design; (d) presented original data; (e) tested members of the general population; (f) *N* ≥ 5; (g) included virtual environments that aimed to promote or measure relaxation or relaxation-related variables; and (h) presented immersive and interactive, three-dimensional virtual environments in HMD. Papers were excluded if they (a) tested a clinical population; (b) targeted specific anxieties or anxiety disorders; or (c) presented virtual environments in two-dimensional graphics on screens or caves.

### Quality assessment

Quality ratings of included studies were carried out by two independent reviewers (LA, SP) using the Effective Public Health Practice Project (EPHPP) quality assessment tool for quantitative studies [18]. Quality rating were calculated in consultation with other researchers (SR, LB). EPHPP rates six methodological domains: selection bias, study design, confounders, blinding, data collection, and withdrawals. A global rating for each study is calculated as: ‘strong’ = no weak subscale ratings; ‘moderate’ = one weak subscale rating; ‘weak’ = two or more weak subscale ratings.

## Results

### Study characteristics

**Fig. 1:**
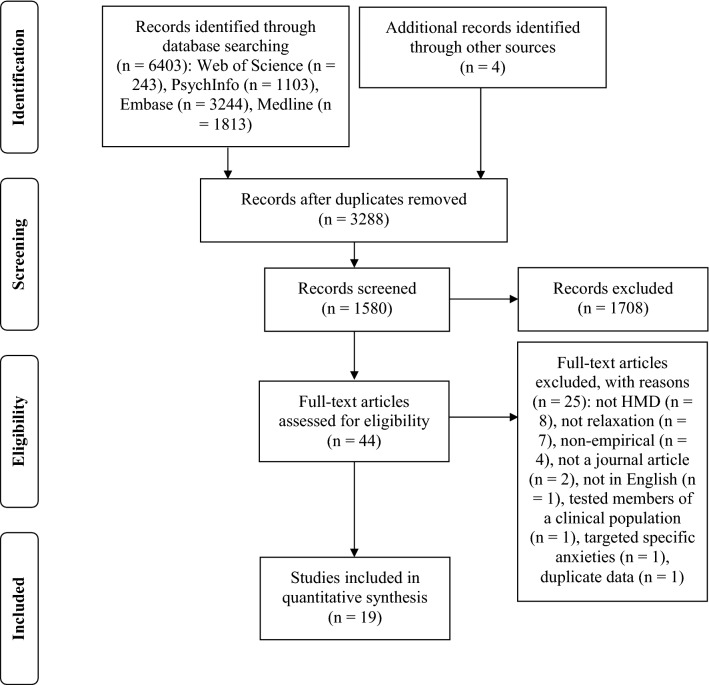
Preferred Reporting Items for Systematic Reviews and Meta-Analyses (PRISMA) flow diagram of studies on virtual reality relaxation for the general population

A total of 6403 articles were identified through database searching and four articles through other sources. The full texts of 44 studies were screened and, of these, nineteen met inclusion criteria and were included in the review. See Fig. 1 for the Preferred Reporting Items for Systematic Reviews and Meta-Analyses (PRISMA) flow diagram. See Table 1 for study characteristics. The nineteen included studies were published between 2007 and 2020. There were thirteen controlled studies, of which four were randomised controlled trials (RCTs). Studies were conducted in USA (*N* = 3), Germany (*N* = 3), Australia (*N* = 3), Italy (*N* = 3), China (*N* = 3), Spain (*N* = 2), Belgium (*N* = 1), and Canada (*N* = 1). A total of 1,278 participants took part in the studies, of which 662 participants experienced an HMD intervention. The number of participants recruited across studies ranged from sixteen to 190. Two studies had sample sizes over 100 [19, 20]. Eleven studies used student participants. Most participants across studies were in the 20–40 age range. Eighteen studies contained one intervention session and one contained four intervention sessions, with the duration of each session ranging from three minutes to one hour. Follow-up sessions were reported in two studies and took place two weeks [21] or one month and three months [22] following the initial intervention. HMDs used in studies were Oculus Rift (*N* = 8), Oculus Go (*N* = 2), Samsung Gear (*N* = 2), Sony Glassroom (*N* = 2), Pico Goblin (*N* = 1), nVIS (*N* = 1), and HTC Vive (*N* = 1). Two studies included no information on HMD branding. Of these, one reported the inclusion of second-generation VR glasses of the illusion mirror type [23] and another outlined 800 × 600 resolution VR HMD with head tracking [24].

**Table 1:** Characteristics of studies on virtual reality relaxation for the general population

Study	Country	*N*	Experimental group (*N*)	Comparison group (*N*)	Mean age (SD)	Apparatus	Experimental condition	Comparison condition(s)	Measures	Sessions	Follow-up	Findings
Anderson et al. [33]	USA	18	18 (9F)	None	32 (12)	HMD (Oculus Rift DK2, Oculus VR, Menlo Park, CA); Ireland VR from http://www.feeltherelaxation.com; Dream Beach VR, from http://www.feeltherelaxation.com; HR device (Biopac MP150 with EDA100C and ECG100C modules, Goleta, CA). AcqKnowledge 4.4.0, Biopac software; beach lounge chair, heat lamp	Experience natural scenes from Ireland including water/animals/ evidence of human presence (houses/roads), and Australian beaches with no animals/no evidence of human presence, ocean sounds and music, physically immersive with beach lounge chair and heat lamp (15 min per scene)	Experience an empty classroom with no people/plants/animals (15 min)	VVR; EDA; EKG; HRV; PANAS; MRJPQ; qualitative comments on scene; rating of favourite to least favourite scene	1	None	Natural scene in HMD increased relaxation objectively and subjectively. Scene preference had a significant effect on mood and perception of scene quality
Browning et al. [26]	USA	190; 82 completed measures (39F)	30 nature exposure in VR group	30 nature outdoors group; 30 without nature	20 (1.2)	015 Samsung Gear VR headset with a Galaxy Note 5 smartphone; Audio-Technica ATH-ANC7B QuietPoint Active Noise-Cancelling Closed-Back headphones; Samsung Gear 360 camera; Zoom H1 external microphone. Gear 360 ActionDirector (Samsung, Seoul, South Korea)	Watch 360-degree video and soundscape of outdoor forest setting with songbirds, small mammals and moderately dense foliage (6 min)	Experience real outdoor forest with birds, small mammals and foliage (6 min); sit in front of a blank wall in indoor setting with no visual/auditory access to nature (6 min)	PANAS; PRS; DSS; EBS-NB; no. of times used VR on 6-point scale; nature exposure on 9-point scale; demographics; SCL	1	None	HMD nature exposure preserved positive affect, outdoor nature exposure increased positive affect, no nature exposure diminished positive affect. Both nature exposures increased physiological arousal showing benefits beyond variance explained by preference/nature/HMD experiences/demographic characteristics
Cebolla et al. [21]	Spain	16	8 guided meditation with VR (5F)	8 guided meditation with no VR (7F)	30.56 (10.86)	The Machine to be another (TMTBA); HMD (Oculus Rift); camera	“Performer” facilitates a 3rd person perspective of the participant by wearing a camera and mirrors participant movements. Participant is invited to view themselves via TMTBA; perform movements in embodiment induction (5 min); listen to guided compassion-based meditation (CBM) with HMD turned off (15 min); view self and listen to self-compassionate messages, hug “self”(“body swap condition” 5–7 min)	Listen to guided mediation (15 min)	Socio-demographics; Psychological and Practice-Related Meditation Variables Questionnaire; PHQ-9; GAD-7; QMI; PANAS; SMS; SOFI; MSCS; Adherence Questionnaire; Embodiment in TMTBA Likert scales	1	2 weeks later	CBM with/without HMD significantly increased positive qualities toward self/others, decreased negative qualities toward self, increased awareness and attention to mental events and bodily sensations. No differences between conditions. At follow-up, both conditions showed similar frequency of meditation practice. Frequency of clinical self-care behaviours significantly higher in TMTBA. Lower imagery ability in visual and cutaneous modality moderated efficacy of TMTBA (vs. no HMD) in increasing adherence
Gao et al. [19]	China	120 (62F)	20 in each condition divided by 6 types of environment	None	20.7 (2.13)	Panoramic camera (Insta360 Pro-I). Photographs with resolution of 7680 × 3840 (8 K) pixels. VR glasses (Pico Goblin VR all-in-one) with resolution of 2560 × 1440 pixels, and screen refresh rate of 70 Hz (< 20 ms); NeuroSky portable brainwave device with a NeuroSky TGAM brain wave chip	30 panoramic photos of urban environments in China divided into six categories (grey space/ blue space/open green space/ partly open green space/partly closed green space/closed green space). Observe 5 panoramic photos of allocated environment category (5 min)	None	EEG; POMS-S; Stroop colour task; environment preference ratings	1	None	HMD showed restorative effects on attentional fatigue and negative mood. Partly open green space had the most significant effect on negative mood regulation. Strong positive correlation between preference for environment and improvement of positive mood
Liszio et al. [34]	Germany	62 (36F)	22 in VR HMD group	17 in desktop group; 23 with no distraction	22.6 (5 .36)	Audiovisual VR underwater simulation (“theBlu”, Wevr, 2016); 17″ screen or HMD (Oculus Rift CV1); built-in headphones or desktop speaker; commercial heart rate monitor and chest belt	Watch underwater environment (7 min)	Sit and watch the same underwater environment via desktop (7 min); or wait with no distraction (7 min)	HRV; salivary cortisol; STAI; PANAS; IPQ; GEQ; VR-TSST	1	None	HMD underwater scenario reduces physiological stress, anxiety and negative feelings. Significantly higher HRV levels (i.e. less stress) in HMD than desktop and CG. Participants using HMD had lower subjective anxiety levels than desktop/CG, and less negative affect than CG. Perceived immersion impacts anxiety directly
Liszio et al. [37]	Germany	57 (41F)	19 in interactive group; 19 in non-interactive group	19 with no distraction	23.7 (5.67)	OculusRift and Touch controllers; heart rate monitor and chest belt; VR app developed by authors	In interactive group, play two games including throwing coconuts on wooden barrels and feeding flowers to turtles (9 min). In non-interactive group, observe beach with butterflies, turtles, and natural background noises (9 min)	Wait for experiment to continue (9 min)	HRV; PANAS; STAI; IPQ; SSQ; VR-TSST	1	None	Relaxation and positive affect significantly increased in interactive HMD condition compared to non-interactive and CG
Liu et al. [27]	USA	40	40 student athletes	None	18–25 (range; no mean reported)	WORLDS VR environments created for relaxation and mental wellness by IFGworld™ (Los Angeles, CA); HMD (Oculus Go)	Choose from 9 indoor and outdoor settings including a beach, bamboo forest, an artist’s loft, a teahouse. Switch between environments as desired (5–15 min)	None	Novel survey about perceived relaxation and VR experience (yes/no responses); QLQ	1	None	Participants found HMD relaxing and would use again. Most participants believed using HMD before competition would help to relax them. Relaxation could still be attained with motion sickness
Navarro-Haro et al. [32]	Spain	44	44 (28F)	None	45.32 (13.20)	HMD (Oculus Rift DK2); Dr. Linehan’s DBT^®^ Mindfulness Skills™ audios (http://behavioraltech.org); MSIGT Series GT72 Dominator ProG-1252 Gaming Laptop 6th Generation Intel Core i7 6700HQ (2.60 GHz) 16 GB Memory 1 TB HDD 512 GB SSD NVIDIA GeForce GTX 980 M 4 GB GDDR5 17.3" Windows 10 Home 64-Bit; Bose Q25 headphones; Visuals created/ copyrighted by BigEnvironments.com (Unity 3D software)	Listen to one session of DBT mindfulness skills training instructions (either wise mind/ observing sound/ observing visual) while floating down a river with ripples, trees, boulders and mountains (10 min)	None	Demographics; meditation frequency; emotions VAS; MAAS; adaptation of Sense of Presence Questionnary, adaptation of CEQ, adaptation of ITC-SOPI	1	None	After HMD, participants reported subjective improved mindfulness state and reduced negative emotional states. Participants reported significantly less sadness, anger, and anxiety, being significantly more relaxed, moderate to high “presence” in HMD, and showed high acceptance of HMD as a technique to practice mindfulness
Naylor et al. [29]	Australia	49	16 (5F) in ‘SoundSelf’ condition	16 (10F) in breathing condition; 17 in ‘Rainy day’ condition (6F)	27.33 (6.96)	Oculus^®^ Rift™ Development Kit 2 (Oculus VR, LLC, Menlo Park, CA, USA); Windows 7 laptop; ‘SoundSelf’ condition: 20-min of meditation program (SoundSelf alpha build 2015–06-10); Breathing condition: audio from guided breathing exercise (Cura smile YouTube channel (2013) “relaxation breathing guided”, 20 min), visuals generated using VisiR (version 0.7.5) from Valynx Studio (2015)); control condition: “Rainy Day Office Window” video (Jason Comerford Photography YouTube channel (2015), 20 min); Fitbit® Charge HRTM (Fitbit Inc., San Francisco, CA, USA)	‘Soundself’ condition: Look around a void of colourful lights and sounds with interactive and immersive features (20 min)	Breathing condition: look around void of colourful lights and sound with graphic visuals changing colour/pattern in response to paired audio with no interactive features (20 min); ‘Rainy day condition’: Watch 2D screen with tree and leaves on rainy day with streetscape background (20 min)	Demographics; PANAS; Stroop Test; HR; Likert scale questions; qualitative questions	1	None	All HMD conditions decreased HR significantly and participants reported increased relaxation after HMD. After HMD, positive and negative affect reduced, suggesting increased lethargy. Breathing condition showed significant interaction between time and condition on negative affect. Participants reported feeling relaxed, identified influencing factors, and supported HMD in workplace/college
Riva et al. [24]	Italy	61 (35F)	61	61	21.45 (2.91)	PC (Sony Vaio Notebook PCG-GRT 996ZP, Pentium-4 3.20-GH) with Microsoft Windows XP Professional and graphic card (NVIDIA GeForce FX Go5600 with 3D performance), 64 MB of VRA; HMD (800 × 600 resolution and head tracking); Logitech Wingman Cordless Rumblepad Gamepad; two amplifiers and speakers (Star SP-160B)	Park with trees, lamps, summer cinema, band stand etc. Relaxing experience associated to the park by manipulating sound and music, shadows, lights and textures. Explore park while answering emotion and presence rating questions (3 min per park)	2 parks with trees, lamps, summer cinema, band stand etc. Anxious or neutral experience associated to park by manipulating sound and music, shadows, lights and textures. Explore parks while answering emotion and presence rating questions (3 min per park)	BDI; VAS; PANAS; STAI; UCL-PQ; ICT-SOPI; emotion and presence ratings scale 1–10	1	None	HMD environments produced anxiety and increased relaxation effectively. The feeling of presence was greater in the “emotional” environments; emotional state was influenced by level of presence
Rockstroh et al. [31]	Germany	94	94 randomly assigned to 4 conditions (64F)	None	23.8 (4.9)	Computer screen (Dell U2415); HMD (Oculus Rift CV1); biofeedback equipment BITalino(r)evolution board. Adhesive Ag/AgCl electrodes	Relax and observe forest and changing weather and soundscape using HMD either with, or without biofeedback (10 min)	Relax and observe forest and changing weather and soundscape using 2D screen either with or without biofeedback (10 min)	IPQ; PRS; STAI; EDA	1	None	EDA and perceived stress reduced across all conditions. Display type and biofeedback did not show significant differences in relaxation. HMD increased presence compared to screen. HMD with biofeedback increased physical presence. HMD and biofeedback increased elements of perceived restorativeness
Schutte et al. [28]	Australia	26	26 randomly assigned to natural or urban environment (16F)	None	34.46 (12.60)	Samsung 360-degree panoramic camera; Samsung360 VR headset (panoramic 360 head-tracking with corresponding directed sound); swivel chairs	Experience natural environment in Australia (eucalyptus tree/meadow/ stream with sound of birds/running water), or urban environment in small Australian town (buildings/road traffic/pedestrian mall/ sound of traffic/talking)	None	PANAS; PRS; CNS	1	None	Natural environment increased positive affect and perceived restorativeness. Restorativeness mediated the relationship between environment and positive affect
Seabrook et al. [35]	Australia	40 (37 completed measures)	37 (24F)	None	37.86 (14.56)	Camera (Z CamV1 Pro, 4 K resolution, height set at 1.3 m); omnidirectional and stereo microphones (Zoom H6 and Zoom H2n); HMD (Oculus Go with hand controller); VR mindfulness app; swivel chair	Explore environment and listen to guided mindfulness exercise. 2 forest scenes in Great Otway National Park, Australia with ambient sounds. Site 1 is a clearing next to a river, site 2 at river’s edge. No people or animals (15 min)	None	Demographics; FFMQ-15; DASS-21; SMS; SSQ; PQ; emotions Likert ratings; Qualitative feedback from 19 participants	1	None	State mindfulness and positive affect significantly increased after using VR app. No changes in negative emotion, subjective arousal, or simulator sickness. Participants described experience as relaxing, calming, and peaceful
Valtchanov et al. [25]	Canada	22	12 (6F)	10 (6F)	17–26 (range; no mean reported)	High-resolution HMD (nVIS, Reston, VA) with 65-degree view and light-blocking cover; InertiaCube Tracker (InterSense Inc., Billerica, MA); rumble platform; computer; PowerLab Data Acquisition System(ADInstruments, Colorado Springs, CO); LabChart software; fingertip electrodes and sensors; ElderScrolls IV: Oblivionworld construction set (Bethesda Soft-works LLC, Rockville, MD; Elder Scrolls IV: Oblivion engine using a GeForce 8800(nVIDIA, Santa Clara, CA) at 1280 × 1024 resolution per eye; wireless mouse; stereo headphones	Explore rain forest with shrubs, flowers, trees, streams, ponds, and varied rock and terrain levels. Ambient natural sounds played and autonomous movement of environment. “Forest Breeze” Wick Air freshener used for scent (10 min)	Watch slide show of 10 abstract paintings with colours commonly found in nature (lots of green and blue with some yellow, orange and red) in virtual dark room (10 min)	ITQ; SCL, ZIPERS score; HR; mental arithmetic score	1	None	Nature HMD significantly decreased SCL and significantly increased positive affect. No differences between HMD and controls shown for negative affect
Van Kerrebroeck et al. [20]	Belgium	183	103	80	34.7 (13.7)	Oculus Rift DK2; headphones; Christmas-themed props and ornaments	Pretend to be Santa Claus riding sleigh being pulled by reindeer through snowy Nordic landscapes. Participants can see Santa’s belly when looking down (3 min)	Control environment was exposure to same mall as VR group with Christmas decorations. No task	Perceived crowdedness ratings; Big 5 personality scale; optimum stimulation level rating; allotted time to shopping trip; items on escapism, presence, relaxation and pleasure; items on attitudes to mall; mall satisfaction and loyalty intentions	1	None	Participants exposed to HMD showed better attitude towards mall, increased mall satisfaction and loyalty compared to controls, and experienced high level of presence, pleasure, escapism, and relaxation. VR can mitigate the effects of perceived crowdedness
Villani et al. [36]	Italy	64 (34F)	16 in VR group	16 in DVD group; 16 in audio group; 16 no treatment	24.52 (1.75)	Relaxation Island VR programme; PC (Fujitsu Siemens AMILO Processor, Pentium 4); wireless joystick (Logitech Wingman Cordless Rumblepad Gamepad); HMD (Sony Glasstron PLM S-700 with a head-tracker, Intersense Intertrax2); audiotape with headphones. For physiological measurements: BioGraph Infiniti Procomp; swivel armchair	Relaxing island with four zones related to different relaxation exercises: waterfall, clouds, beach1, and beach2. Listen to relaxation narrative	Listen to relaxation narrative while watching DVD with four beaches of tropical islands; or listen to audiotape only; no treatment	STAI; VAS; PANAS; COPE; ITC-SOPI; HR, Ham, Rr, Ram, Sc, Em	1	None	HMD, DVD and audio significantly reduced anxiety and significantly increased positive emotional states including relaxation. Sense of presence mediates relationship between media type and effectiveness of intervention
Villani et al. [22]	Italy	36	12 in HMD group; 12 in video group	12 in audio group	18–35 (range); M = 25.21 (1.44) F = 25 (0.87)	HR device (BioGraph Infiniti Procom); 3D Game Studio of Conitec software; two photographs [lake stimulus number 5780 of the International Affective Picture System (IAPS); garden stimulus number 5760 of the IAPS]; PC (Fujitsu Siemens AMILO Processor, Pentium Core 2 Duo with an ATI Radeon HD3450, 512 Mb, graphic card); wireless joystick (Logitech Wingman Cord-less Rumblepad Gamepad); HMD; SonyGlasstron PLM S-700; audiotape with headphones (Sony MDR-EX51LP Fontopia in-the-ear headphones)	HMD condition: Wilderness park with ‘natural zones’ (lake, river, waterfall, garden, forest). Listen to narrative and interact/explore environment; Video condition: Watch video of the same wilderness park via HMD and listen to narrative with no interaction	Audio condition: Listen to narrative guiding through scenario and use imagination to contextualise the experience	MPS; STAI; HR, SCL; respiration	4 (1 assessment, 3 with either HMD, video or audio)	After 1 month; after 3 months	All media types induced significant changes in HR and anxiety state level in guided sessions and follow-up. Participants in HMD condition were better at reducing HR and significantly improved emotional state during experimental and follow-up sessions
Wang et al. [23]	China	96 (63F)	96 assigned to one of 7 environment types: (1) structure: 15; (2) wood:13; (3) wood with bench: 12; (4) wood with platform and bench: 14; (5) platform with trees: 12; (6) waterfall with trees: 15; (7) pool with plants: 15	None	24.03 (5.29)	UCVR EYE-01 camera (Pinkang Smart Company, Changzhou, China); BP and HR monitor HEM-7111 electronic sphygmomanometer (upper arm, OMRON, Dalian, China); saliva collection tube (salivette, SARSTEDT, Sarstedtstraße, Germany); second-generation VR glasses of the illusion mirror type	Participants asked to imagine themselves in one of seven forest resting environments in Beijing forest parks: (1) structure, (2) wood, (3) wood with bench, (4) wood with platform and bench, (5) platform with trees, (6) waterfall with trees, (7) pool with plants (5 min each)	None	BP, HR, salivary amylase; BPOMS	1	None	All environments reduced stress. Types 1 and 4 (more artificial structures) had significantly different effects on physiological stress relief compared to 4, 3, 6 and 7 (more natural and wood features), but artificial structures increased psychological recovery. Environment containing some facilities provided better stress relief than pure natural environment. Dynamic water landscape facilitated stress relief
Yang et al. [30]	China	60	30 (16F)	30 (18F)	19–23 (range; no mean reported)	Colour pencils/brushes/erasers, 2D human body model printed on paper (control condition); HMD (HTC Vive system with resolution of 1080 × 1200 pixels, with frame refresh rate of 90 Hz); two wireless controllers; two base stations	Using HMD with drawing tools/colours/textures/sounds, design a wearable technology product that could perform/refine smartphone functions on 3D human model (5 min)	Using paper and pencil, design wearable technology product that could perform/refine smartphone functions (5 min)	EEG; FSS; K-DOCS; expert panel scale to assess creative quality	1	None	Participants with HMD maintained more stable focus/attention. Participants in paper and pencil condition were more relaxed. HMD designs were rated as having higher creative quality than paper and pencil
Total participants		1278	735	401								

KEY: ***Demographics***: *M* Male, *F* Female; ***Conditions:*** *CG* control group, ***Equipment:*** *HMD* head-mounted display; ***Measures****:* *BDI* Beck depression inventory, *BP* blood pressure, *BPOMS* Brief profile of mood states, *CEQ* Credibility/Expectancy Questionnaire, *CNS* Connectedness to Nature Scale, *COPE* Coping Orientation to Problems Experienced Questionnaire, *DASS* Depression and Anxiety Stress Scale, *DSS* Disgust Sensitivity Scale, *EBS-NB* Engagement with Beauty Scale-Natural Beauty Subscale, *EDA* electrodermal activity, *EEG* electroencephalogram, *EKG* electrocardiogram, *Em* electromyographic responses, *FFMQ-15* Five Facet Mindfulness Questionnaire, *FSS* Flow State Scale, *GAD-7* Generalised Anxiety Disorder Questionnaire, *GEQ* Game Experience Questionnaire, *Ham* heart amplitude, *HR* heart rate, *HRV* heart rate variability, *IPQ* Igroup Presence Questionnaire, *ITC-SOPI* Independent Television Company Sense of Presence Inventory, *ITQ* Immersive Tendencies Questionnaire, *K-DOCS* Kaufman Domains of Creativity Scale, *MAAS* Mindfulness Attention Awareness Scale, *MRJPQ* Modified Reality Judgment and Presence Questionnaire, *MPS* Mesure du Stress Psychologique, *MSCS* Mindfulness Self-Care, *PANAS* Positive and Negative Affect Schedule, *PHQ-9* Patient Health Questionnaire-9, *POMS-SF* Profile of Mood States-Short Form, *STAI* State-Trait Anxiety Inventory, *PRS* Perceived Restorativeness Scale, *PSS* Perceived Stress Scale, *QMI* Betts’ Questionnaire Upon Mental Imagery, *SMS* State Mindfulness Scale, *QLQ* Quality of Life Questionnaire, *Ram* respiration amplitude, *Rr* respiration rate, *RRS* Relation Rating Scale, *Sc* skin conductance, *SCL* skin conductivity levels, *SOFI* Self-Other Four Immeasurable Scale, *SOPQ* Sense of Presence Questionnaire, *SSQ* Simulator Sickness Questionnaire, *UCL-PQ* UCL-Presence Questionnaire, *VAS* visual analogue scale, *VVR* Value of Virtual Reality Questionnaire, *VR-TSST* VR-Trier Social Stress Test

Ten studies included virtual environments consisting only of nature-related stimuli, such as trees, foliage, water, animals, clouds, lakes, rivers, beaches, forests, rocks, and terrain elevations. The RCTs displayed forest or wilderness scenery with lakes, rivers, waterfalls, gardens [22], shrubs, trees, flowers, rocks, terrain elevations [25], vegetation, water, and wooden structures [23]. Six studies included both natural and urban elements in the virtual environments [19, 20, 24, 26–28]. Studies with larger sample sizes included virtual environments consisting of a combination of nature and urban-related stimuli such as sleighs, reindeers, snow [20], public spaces, and greenery [19]. Of these six studies, three compared natural virtual environments with urban virtual environments [19, 27, 28]. Two studies used guided meditation combined with audio-visual features [21, 29], and one applied a drawing activity on a three-dimensional human-like model [30].

Thirteen studies explicitly specified relaxation as a primary outcome variable, among other outcome variables. Measures, such as heart rate variability and skin conductivity levels, were employed to assess relaxation scores pre- and post-intervention. Six studies measured relaxation indirectly through relaxation-related variables such as restoration [19, 26, 28, 31] and stress [23, 25].

### Quality assessment

Six studies received a global rating of ‘strong’, seven were ‘moderate’, and six were ‘weak’. Of these, two studies lacked clarity and detail on study design, nine on confounding variables, and eight on data collection method. See Table 2 for quality ratings. Selection bias was moderate in fourteen studies given that participants were considered representative of the general population. Study designs of thirteen studies were strong because participants were randomly allocated to conditions. Of these, four were RCTs and nine were classified as controlled clinical trials because no method of randomisation was described. Controlling of confounding variables was rated as strong in ten studies. Blinding was moderate in all studies because they lacked information on whether outcome assessors were aware of the intervention or exposure status of participants and whether participants were aware of research questions. Data collection tools were strong in eleven studies. Withdrawals and drop-outs were strong in all studies due to completion rates between 80 and 100%.

**Table 2:**
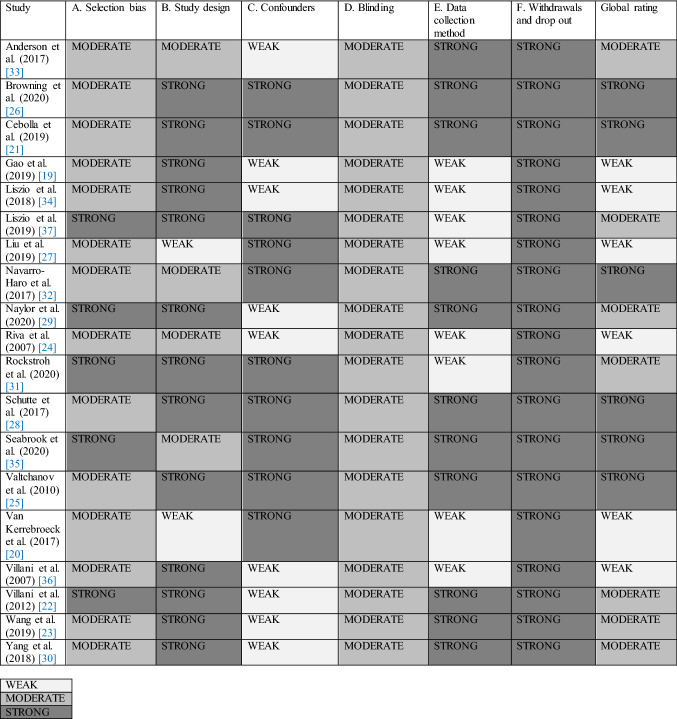
Quality ratings of studies on virtual reality relaxation for the general population

### Feasibility

Nine of the nineteen studies explicitly evaluated feasibility. All indicated that VR is feasible to support and promote relaxation. Of these, five studies indicated that VR is cost-effective and is becoming widely accessible to the general public [22, 25, 26, 29, 32]; however, this mainly consisted of commenting on the cost-effectiveness of VR rather than using primary data to support their claims. Two RCTs indicated that the general population can benefit from the availability and affordability of VR to help alleviate and manage stress [22, 25]. One study with a large sample (*N* = 183) found that in stressful or chaotic situations, such as crowded shopping areas, VR can be a practical way to facilitate relaxation and enjoyment [20]. Two studies indicated that VR is a convenient and easy-to-use tool that supports wellbeing in the general population, particularly for those who have limited time [33] or cannot access the restorative benefits of nature [34]. Two studies reported minor limitations with regard to feasibility: in one study, a small number of participants reported physical difficulties with wearing the HMD [29] and another study reported that the weight of the HMD needs to be tolerable for VR relaxation to be feasible [35].

### Acceptability

Six of the nineteen studies explicitly evaluated acceptability of VR relaxation. Of these studies, five supported the acceptability of HMD relaxation [27, 29, 32, 33, 35] and one study reported inconclusive results [34]. In general, participants found VR relaxation interventions positive, enjoyable, valuable [33], calming, and peaceful [35]. Studies reported that 80% of participants would recommend VR relaxation to manage stress [29] and 90% of participants wished to experience VR relaxation again [27]. VR relaxation was found to be a highly useful tool to support mindfulness practice [32]. Acceptability was less clear in one study that included a virtual simulation of an underwater environment [34]. Although experiencing the virtual underwater environment was enjoyable for most participants, a small number felt uncomfortable due to the “open water” or expressed concerns about sea creatures.

### Effectiveness

Thirteen of the nineteen studies measured relaxation as a primary outcome. The main measurement tools used to assess relaxation were heart rate, self-report questionnaires such as the Positive and Negative Affect Schedule or the State-Trait Anxiety Inventory, or visual analogue scales on perceived relaxation. Ten studies reported increased relaxation in VR conditions, two found increased relaxation in HMD and comparison conditions of 2D graphic visuals [29] and DVD or audio [36], and one found increased relaxation in the comparison condition of a 2D drawing task only [30]. Two RCTs with follow-up data found that relaxation increased in HMD experimental conditions, compared to comparison conditions [21, 22]. Of these, one study showed that meditation combined with embodied VR increased the frequency of mindful relaxation compared to the comparison condition of meditation without embodied VR [21]. When compared to 2D video or audio, one study found that a wilderness virtual environment combined with relaxation exercises and a relaxing narrative in VR produced increases in relaxation [22]. In terms of follow-up data, these studies found that participants in HMD conditions experienced increases in the frequency of clinical self-care behaviours two weeks following the initial intervention [21] and were better at reducing their heart rate level and improving their emotional state one month and three months following the initial intervention [22]. One study with a large sample (*N* = 183) found that participants in a shopping centre exposed to a snowy, Christmas-themed virtual environment experienced increased relaxation [20]. In terms of relaxation-related variables, four studies reported increases in restoration [19, 26, 28, 31] and two reported decreases in stress [23, 25] in HMD conditions. One study with a large sample (*N* = 120) included virtual environments varying in urban space and vegetation and showed that these increased restoration in terms of improving directed attention and negative mood [19]. Another study reported greater increases in positive mood, as well as relaxation, in interactive HMD condition compared to non-interactive HMD and wait-list comparison conditions [37].

## Discussion

### Summary of findings

This systematic review aimed to synthesise current evidence on feasibility, acceptability, and effectiveness of VR relaxation in the general population. Nineteen studies were included in the review, of which four were RCTs. VR was shown to be a feasible and acceptable tool to promote relaxation. Virtual environments with pleasant, often natural, stimuli improved relaxation and relaxation-related variables, such as restoration and stress, when compared to comparison conditions.

These findings are consistent with research indicating that VR is more assessable and more affordable to members of the general public than it has ever been [33, 38], while it is important to recognise that this technology remains prohibitively expensive to many individuals and that social inequalities may contribute to a digital divide [22, 25, 39]. Although practical issues and safety concerns, such as the weight of the headset, were highlighted by a minority of participants [29, 35], recent developments in HMD have ensured that headsets are lightweight and comfortable [11]. High levels of acceptability found in this review are consistent with the view that HMD is a safe tool to support mental health and wellbeing [40].

In most studies included in this review, relaxation scores were significantly higher in HMD experimental conditions than in comparison conditions. This is consistent with previous research that indicates that pleasant and immersive virtual environments support and promote relaxation [41], stress restoration [42], and positive mood [43]. Many of the studies that reported increases in relaxation included audio and visuals of nature, which is consistent with existing research on the effectiveness of nature-related stimuli in facilitating stress recovery [12]. The combination of natural audio-visual features in virtual environments has been shown to activate the parasympathetic system and facilitate relaxation, stress recovery, and mood regulation [25, 44]. Therefore, experiencing natural virtual environments in VR is a promising alternative to obtaining the restorative effects of contact with real-world nature, especially for people who may be unable to access nature or outdoor environments.

### Strengths and limitations of studies included in the review

Strengths of the studies included the prevalence of control or comparison conditions, which enabled researchers to isolate and attribute changes in outcome variables to VR relaxation, and the employment of both physiological and psychological measures. Limitations included the prevalence of young adult and student samples which means that findings may not generalise to other age groups or people of lower education, the relatively small sample sizes, the limited number of sessions, and the lack of follow-up data. Greater numbers of sessions and follow-ups are fundamental to ascertain if positive effects can be maintained. Without this longitudinal data, it is unclear whether there are any sustained or longer-term benefits. Studies were subject to various forms of bias. For instance, reliance on researcher-administered self-report measures may have led to more favourable reporting by participants, and the prevalence of the single-session format may have led to a novelty bias, in that positive evaluation might be attributable to the novelty of VR rather than the intervention itself. Overall, the methodological quality of the studies varied. While most studies stated that participants were randomly allocated to conditions, only four studies described the method of randomisation employed and were classified as RCTs.

### Strengths and limitations of the review

This is the first review to focus on the feasibility, acceptability, and effectiveness of VR relaxation for the general population. The methodology included formal searches on electronic academic databases, as well as non-indexed searching of reference lists. The methods increased the number of studies identified and strengthened the confidence that conclusions arising from this review can be based on the synthesis of all relevant and available research. Screening, data extraction, and quality ratings were completed by two independent researchers, which ensured for an accurate and objective process.

A key limitation of this review is that heterogeneity of concepts limited comparisons between studies and reduced the reliability of findings that were synthesised. In particular, the operational definition of ‘relaxation’ in previous research is broad and comprises multiple facets. The current review identifies a lack of consistency and standardisation of definitions, measures, and interventions of relaxation across the studies reviewed. Although some studies stated relaxation as a primary outcome measure, definitions of relaxation were varied, with some inconsistencies, and there were no formal, validated measures of relaxation. Instead, studies employed physical parameters, idiosyncratic self-report measures, or psychometrics of relaxation-related variables. Single items on perceived relaxation were sometimes included in self-report measures; however, there was no stand-alone measure of relaxation. Similarly, virtual environments intended to promote relaxation were diverse. While most studies used natural virtual environments, other studies combined both natural and urban features. As a result, caution is needed when comparing studies, and the conclusion that nature-based virtual environments are effective to promote relaxation should be stated tentatively.

### Applications to improve wellbeing

The finding that VR relaxation improves relaxation and relaxation-related variables, such as stress, in the general population indicates that VR may be a useful tool to promote relaxation in the home and workplace. Existing studies have highlighted the benefits of VR relaxation and stress management in worker populations highly exposed to stress [45]. This, together with studies included in this review, indicates the potential value of VR to aid the public in managing and preventing cumulative stress. In line with a recent scoping review, studies reviewed indicate that HMD with natural virtual environments is a feasible and acceptable strategy that can be integrated into stressful and demanding situations, such as workplace settings, to improve relaxation and stress levels [46]. Previous studies have established the mental health benefits of VR relaxation in key workers, such as healthcare professionals, experiencing high levels of work-related stress and burnout [47, 48]. Consistent with the studies included in this review, research has shown that workers feel more relaxed after experiencing VR relaxation and respond favourably to the implementation of VR relaxation interventions at work [49].

VR relaxation may have significant public health benefits during the COVID-19 pandemic. This is an unprecedented and hugely challenging situation that has elevated the rates of stress and fear in societies worldwide [50]. Emotional and socio-economic instabilities have been suggested to account for this [51, 52]. Due to government guidelines of social distancing, remote working, and self-isolation in lockdowns, a large proportion of the population are confined to their homes with limited social interaction, which may negatively impact on mental health, particularly in vulnerable groups [53], and the potential for virtual natural environments to support people who cannot experience real-world nature could be significant for both home use and the remote interventions facilitated by health services. For instance, VR relaxation could be trialled as a low-intensity intervention in mental health services. Systematic reviews have established VR as an effective treatment for a range of mental health problems, such as anxiety disorders and psychosis [10]. Extensive evidence suggests that the implementation of VR in clinical settings improves coping strategies [54], safety-seeking behaviours [54], sense of presence [55], and social cognition [40, 56, 57] in mental health service users. VR relaxation could also benefit service users in psychiatric wards where they may be experiencing high levels of stress [58].

### Future research

Future research should aim to standardise definitions, measures, and interventions; and consider demographic and social differences within participants. Larger-scale RCTs and longitudinal studies are critical to establishing the effectiveness of virtual environments and clarifying longer-term benefits. The duration of intervention exposure that is optimal in ensuring the feasibility and effectiveness of HMD relaxation remains inconclusive [20] and so should be tested in more robust studies. Future research might test the psychological benefits of natural virtual environments in VR relaxation, but with consideration to the variety of natural stimuli, given that underwater environments may elicit fears or anxieties [34].

## Conclusions

This review is the first to narratively synthesise the literature on VR relaxation in the general population. Most studies combined nature-based virtual environments with soothing sounds or narratives of guided meditation or breathing, and all reported significant increases in relaxation or relaxation-related variables. However, methodological limitations restrict the wider generalisability of findings and any conclusions must be drawn with caution. Nevertheless, VR appears to be a promising tool to facilitate relaxation and stress management in people experiencing high levels of stress; it can be a practical and accessible intervention that enables people to relax at work or at home; and it may have particular relevance in the COVID-19 pandemic given that worldwide stress is on the rise.

## Supplementary Information

Below is the link to the electronic supplementary material.

Supplementary file1 (DOCX 19 KB)

Supplementary file2 (DOCX 18 KB)
