# Supplementary material for: Virtual reality relaxation for the general population: a systematic review
Source: Soc Psychiatry Psychiatr Epidemiol. 2021 Jun 13;56(10):1707–27. doi: 10.1007/s00127-021-02110-z (PMC8197783; doi:10.1007/s00127-021-02110-z)
Supplement: Supplementary file 1 — Supplementary file1 (DOCX 19 KB) [file 127_2021_2110_MOESM1_ESM.docx]

**Supplementary materials 1. Table 3.** Full breakdown of quality ratings of studies on virtual reality relaxation for the general population

| Study | A. Selection bias  Q1. Are individuals selected to participate in the study likely to represent the target population? Very likely = 1, Somewhat likely = 2, Not likely = 3, Can’t tell = 4.  Q2. What percentage of selected individuals agreed to participate? 80-100% agreement = 1, 60-79% agreement = 2, less than 60% agreement = 3, Not applicable = 4, Can’t tell = 5. | B. Study design  Q1. Study design  Randomised controlled trial = 1, controlled clinical trial = 2, cohort analytic = 3, case-control = 4, cohort = 5, interrupted time series = 6, other = 7, Can’t tell = 8. Was the study described as randomised? If yes, was the method of randomisation described? Was the method appropriate? | C. Confounders  Q1. Were there important differences between groups prior to the intervention? Yes = 1, No = 2, Can’t tell = 3. Examples of confounders: race, sex, marital status/family, age, socio-economic status, education, health status, pre-intervention score on outcome measures.  Q2.  Percentage of relevant confounders that were controlled. 80-100% (most) = 1, 60-79% (some) = 2, less than 60% (few or more) = 3, Can’t tell = 4. | D. Blinding  Q1. Was the outcome assessor aware of the intervention or exposure status of participants? Yes = 1, no = 2, Can’t tell = 3.  Q2. Were the study participants aware of the research questions?  Yes = 1, No = 2, Can’t tell = 3. | E. Data collection method  Q1. Were data collection tools shown to be valid? Yes = 1, No = 2, Can’t tell = 3.  Q2. Were data collection tools shown to be reliable?  Yes = 1, No = 2, Can’t tell = 3. | F. Withdrawals and drop out  Q1. Were withdrawals and drop-outs reported in terms of numbers and/or reasons per group? Yes = 1, No = 2, Can’t tell = 3, Not applicable (i.e. one-time surveys or interviews) = 4.  Q2. Percentage of participants completing the study. 80-100% = 1, 60-79% = 2, less than 60% = 3, Can’t tell = 4, Not applicable (i.e. retrospective case-control) = 5. | Global rating |
| --- | --- | --- | --- | --- | --- | --- | --- |
| Anderson et al. (2017) [33] | (Q1) 2. Somewhat likely; (Q2) 1. 80-100% agreement = MODERATE | (Q1) 5. Cohort, randomised. The order of scene presentation was randomised and counterbalanced. This method was appropriate = MODERATE | (Q1) 3. Can’t tell; (Q2) 3. Can’t tell = WEAK | (Q1) 3. Can’t tell; (Q2) 3. Can’t tell = MODERATE | (Q1) 1. Yes; (Q2) 1. Yes = STRONG | (Q1) 4. Not applicable (i.e. one-time surveys or interviews); (Q2) 1. 80-100% = STRONG | 2. MODERATE |
| Browning et al. (2020) [26] | (Q1) 2. Somewhat likely; (Q2) 1. 80-100% agreement = MODERATE | (Q1) 2. Controlled clinical trial, randomised. Participants were randomly allocated to conditions, but method of randomisation was not described = STRONG | (Q1) 1. Yes. Age, gender, race, disgust sensitivity, engagement with beauty, frequency of nature visits, experience with VR, and nervous system’s response to temperature variations; (Q2) 1. 80-100% (most) = STRONG | (Q1) 3. Can’t tell; (Q2) 3. Can’t tell = MODERATE | (Q1) 1. Yes; (Q2) 1. Yes = STRONG | (Q1) 4. Not applicable (i.e. one-time surveys or interviews); (Q2) 1. 80-100% = STRONG | 1. STRONG |
| Cebolla et al. (2019) [21] | (Q1) 2. Somewhat likely; (Q2) 2. 60-79% agreement = MODERATE | (Q1) 1. Randomised controlled trial. Participants were randomly allocated to conditions, using the Random Allocation Software 2.0. This method was appropriate = STRONG | (Q1) 2. No. Age, gender, education level, history of mental or chronic illness, experience with meditation, frequency of meditation, PHQ-9 (depression), and GAD-7 (anxiety); (Q2) 1. 80-100% (most) = STRONG | (Q1) 3. Can’t tell; (Q2) 3. Can’t tell = MODERATE | (Q1) 1. Yes; (Q2) 1. Yes = STRONG | (Q1) 3. Can’t tell; (Q2) 1. 80-100% = STRONG | 1. STRONG |
| Gao et al. (2019) [19] | (Q1) 2. Somewhat likely; (Q2) 1. 80-100% agreement = MODERATE | (Q1) 2. Controlled clinical trial, randomised. Participants were randomly allocated to conditions, but method of randomisation was not described = STRONG | (Q1) 3. Can’t tell; (Q2) 3. Can’t tell = WEAK | (Q1) 3. Can’t tell; (Q2) 3. Can’t tell = MODERATE | (Q1) 3. Can’t tell; (Q2) 3. Can’t tell = WEAK | (Q1) 4. Not applicable (i.e. one-time surveys or interviews); (Q2) 1. 80-100% = STRONG | 3. WEAK |
| Liszio et al. (2018) [34] | (Q1) 2. Somewhat likely; (Q2) 1. 80-100% agreement = MODERATE | (Q1) 2. Controlled clinical trial, randomised. Participants were randomly allocated to conditions, but method of randomisation was not described = STRONG | (Q1) 3. Can’t tell; (Q2) 3. Can’t tell = WEAK | (Q1) 3. Can’t tell; (Q2) 3. Can’t tell = MODERATE | (Q1) 3. Can’t tell; (Q2) 3. Can’t tell = WEAK | (Q1) 4. Not applicable (i.e. one-time surveys or interviews); (Q2) 1. 80-100% = STRONG | 3. WEAK |
| Liszio et al. (2019) [37] | (Q1) 1. Very likely; (Q2) 1. 80-100% agreement = STRONG | (Q1) 2. Controlled clinical trial, randomised. Participants were randomly allocated to conditions, but method of randomisation was not described = STRONG | (Q1) 1. Yes. Simulator sickness; (Q2) 1. 80-100% (most) = STRONG | (Q1) 3. Can’t tell; (Q2) 3. Can’t tell = MODERATE | (Q1) 3. Can’t tell; (Q2) 3. Can’t tell = WEAK | (Q1) 4. Not applicable (i.e. one-time surveys or interviews); (Q2) 1. 80-100% = STRONG | 2. MODERATE |
| Liu et al. (2019) [27] | (Q1) 2. Somewhat likely; (Q2) 1. 80-100% agreement = MODERATE | (Q1) 7. Other, one-time survey and not randomised. Participants only completed a post-intervention survey = WEAK | (Q1) 2. No. Experience with mental wellness apps and motion sickness; (Q2) 1. 80-100% (most) = STRONG | (Q1) 3. Can’t tell; (Q2) 3. Can’t tell = MODERATE | (Q1) 3. Can’t tell; (Q2) 3. Can’t tell = WEAK | (Q1) 4. Not applicable (i.e. one-time surveys or interviews); (Q2) 1. 80-100% = STRONG | 3. WEAK |
| Navarro-Haro et al. (2017) [32] | (Q1) 2. Somewhat likely; (Q2) 1. 80-100% agreement = MODERATE | (Q1) 5. Cohort, randomised. Participants were randomly assigned to different types of training audios in a within-subject design. This method was appropriate = MODERATE | (Q1) 3. Can’t tell. Age, gender, marital status, educational level, employment status, type of work, type of working day, chronic pathology, and meditation frequency; (Q2) 1. 80-100% (most) = STRONG | (Q1) 3. Can’t tell; (Q2) 3. Can’t tell = MODERATE | (Q1) 1. Yes; (Q2) 1. Yes = STRONG | (Q1) 4. Not applicable (i.e. one-time surveys or interviews); (Q2) 1. 80-100% = STRONG | 1. STRONG |
| Naylor et al. (2020) [29] | (Q1) 1. Very likely; (Q2) 1. 80-100% agreement = STRONG | (Q1) 2. Controlled clinical trial, randomised. Participants were randomly allocated to conditions, but method of randomisation was not described = STRONG | (Q1) 3. Can’t tell; (Q2) 3. Can’t tell = WEAK | (Q1) 3. Can’t tell; (Q2) 3. Can’t tell = MODERATE | (Q1) 1. Yes; (Q2) 1. Yes = STRONG | (Q1) 4. Not applicable (i.e. one-time surveys or interviews); (Q2) 1. 80-100% = STRONG | 2. MODERATE |
| Riva et al. (2007) [24] | (Q1) 2. Somewhat likely; (Q2) 1. 80-100% agreement = MODERATE | (Q1) 5. Cohort, randomised. The order of scene presentation was randomised. This method was appropriate = MODERATE | (Q1) 3. Can’t tell; (Q2) 3. Can’t tell = WEAK | (Q1) 3. Can’t tell; (Q2) 3. Can’t tell = MODERATE | (Q1) 3. Can’t tell; (Q2) 3. Can’t tell = WEAK | (Q1) 4. Not applicable (i.e. one-time surveys or interviews); (Q2) 1. 80-100% = STRONG | 3. WEAK |
| Rockstroh et al. (2020) [31] | (Q1) 1. Very likely; (Q2) 1. 80-100% agreement = STRONG | (Q1) 2. Controlled clinical trial, randomised. Participants were randomly allocated to conditions, but method of randomisation was not described = STRONG | (Q1) 2. No. Age, gender, employment status, and arousal level; (Q2) 1. 80-100% (most) = STRONG | (Q1) 3. Can’t tell; (Q2) 3. Can’t tell = MODERATE | (Q1) 3. Can’t tell; (Q2) 3. Can’t tell = WEAK | (Q1) 4. Not applicable (i.e. one-time surveys or interviews); (Q2) 1. 80-100% = STRONG | 2. MODERATE |
| Schutte et al. (2017) [28] | (Q1) 2. Somewhat likely; (Q2) 1. 80-100% agreement = MODERATE | (Q1) 2. Controlled clinical trial, randomised. Participants were randomly allocated to conditions, but method of randomisation was not described = STRONG | (Q1) 1. Yes. Age; (Q2) 1. 80-100% (most) = STRONG | (Q1) 3. Can’t tell; (Q2) 3. Can’t tell = MODERATE | (Q1) 1. Yes; (Q2) 1. Yes = STRONG | (Q1) 4. Not applicable (i.e. one-time surveys or interviews); (Q2) 1. 80-100% = STRONG | 3. STRONG |
| Seabrook et al. (2020) [35] | (Q1) 1. Very likely; (Q2) 1. 80-100% agreement = STRONG | (Q1) 5. Cohort, not randomised. Participants experienced the same condition = MODERATE | (Q1) 2. No. Age, gender, highest level of education, and previous experience with VR and mindfulness; (Q2) 1. 80-100% (most) = STRONG | (Q1) 3. Can’t tell; (Q2) 3. Can’t tell = MODERATE | (Q1) 1. Yes; (Q2) 1. Yes = STRONG | (Q1) 4. Not applicable (i.e. one-time surveys or interviews); (Q2) 1. 80-100% = STRONG | 3. STRONG |
| Valtchanov et al. (2010) [25] | (Q1) 2. Somewhat likely; (Q2) 1. 80-100% agreement = MODERATE | (Q1) 1. Randomised controlled trial. Participants were randomly allocated to conditions. This method was appropriate = STRONG | (Q1) 2. No. Gender and math-quiz difficulty; (Q2) 1. 80-100% (most) = STRONG | (Q1) 3. Can’t tell; (Q2) 3. Can’t tell = MODERATE | (Q1) 1. Yes; (Q2) 1. Yes = STRONG | (Q1) 4. Not applicable (i.e. one-time surveys or interviews); (Q2) 1. 80-100% = STRONG | 3. STRONG |
| Van Kerrebroeck et al. (2017) [20] | (Q1) 2. Somewhat likely; (Q2) 1. 80-100% agreement = MODERATE | (Q1) 8. Can’t tell = WEAK | (Q1) 3. Can’t tell. Age, gender, personality traits, optimum stimulation level (e.g., ‘experience-seekers’), and crowding perceptions (e.g., spatial density, queuing, time pressure or time allotted to shopping trip, shopping motivation); (Q2) 1. 80-100% (most) = STRONG | (Q1) 3. Can’t tell; (Q2) 3. Can’t tell = MODERATE | (Q1) 3. Can’t tell; (Q2) 3. Can’t tell = WEAK | (Q1) 4. Not applicable (i.e. one-time surveys or interviews); (Q2) 1. 80-100% = STRONG | 3. WEAK |
| Villani et al. (2007) [36] | (Q1) 2. Somewhat likely; (Q2) 1. 80-100% agreement = MODERATE | (Q1) 1. Randomised controlled trial. Participants were randomly allocated to conditions, using a true random number service. This method was appropriate = STRONG | (Q1) 3. Can’t tell; (Q2) 3. Can’t tell = WEAK | (Q1) 3. Can’t tell; (Q2) 3. Can’t tell = MODERATE | (Q1) 3. Can’t tell; (Q2) 3. Can’t tell = WEAK | (Q1) 4. Not applicable (i.e. one-time surveys or interviews); (Q2) 1. 80-100% = STRONG | 3. WEAK |
| Villani et al. (2012) [22] | (Q1) 1. Very likely; (Q2) 1. 80-100% agreement = STRONG | (Q1) 1. Randomised controlled trial. Participants were randomly allocated to conditions, using a true random number service. This method was appropriate = STRONG | (Q1) 3. Can’t tell; (Q2) 3. Can’t tell = WEAK | (Q1) 3. Can’t tell; (Q2) 3. Can’t tell = MODERATE | (Q1) 1. Yes; (Q2) 1. Yes = STRONG | (Q1) 3. Can’t tell; (Q2) 1. 80-100% = STRONG | 2. MODERATE |
| Wang et al. (2019) [23] | (Q1) 2. Somewhat likely; (Q2) 1. 80-100% agreement = MODERATE | (Q1) 2. Controlled clinical trial, randomised. Participants were randomly allocated to conditions, but method of randomisation was not described = STRONG | (Q1) 3. Can’t tell; (Q2) 3. Can’t tell = WEAK | (Q1) 3. Can’t tell; (Q2) 3. Can’t tell = MODERATE | (Q1) 1. Yes; (Q2) 1. Yes = STRONG | (Q1) 4. Not applicable (i.e. one-time surveys or interviews); (Q2) 1. 80-100% = STRONG | 2. MODERATE |
| Yang et al. (2018) [30] | (Q1) 2. Somewhat likely; (Q2) 1. 80-100% agreement = MODERATE | (Q1) 2. Controlled clinical trial, randomised. Participants were randomly allocated to conditions, but method of randomisation was not appropriate (if the first person was in the control condition, the next person would be in the experimental condition) = STRONG | (Q1) 3. Can’t tell. Novelty effect of VR environment and individual differences (e.g., interest in task); (Q2) 4. Can’t tell = WEAK | (Q1) 3. Can’t tell; (Q2) 3. Can’t tell = MODERATE | (Q1) 1. Yes; (Q2) 1. Yes = STRONG | (Q1) 4. Not applicable (i.e. one-time surveys or interviews); (Q2) 1. 80-100% = STRONG | 2. MODERATE |

| WEAK |
| --- |
| MODERATE |
| STRONG |
