# Supplementary material for: Virtual reality relaxation for the general population: a systematic review
Source: Soc Psychiatry Psychiatr Epidemiol. 2021 Jun 13;56(10):1707–27. doi: 10.1007/s00127-021-02110-z (PMC8197783; doi:10.1007/s00127-021-02110-z)
Supplement: Supplementary file 2 — Supplementary file2 (DOCX 18 KB) [file 127_2021_2110_MOESM2_ESM.docx]

**Supplementary materials 2. Search strategy**

*Web of Science*

| # 11 | [**243**](https://eur03.safelinks.protection.outlook.com/?url=https%3A%2F%2Fapps.webofknowledge.com%2Fsummary.do%3Fproduct%3DWOS%26doc%3D1%26qid%3D13%26SID%3DD26pnFTNI8xkOpx8KwD%26search_mode%3DCombineSearches%26update_back2search_link_param%3Dyes&data=01%7C01%7Clisa.azevedo%40kcl.ac.uk%7C49146f88507848e1774c08d81c1d75ef%7C8370cf1416f34c16b83c724071654356%7C0&sdata=Xx7INFdrv6iZSIfRp3b8bnKVgXOMrXYQx6KVy4zjJYs%3D&reserved=0) | #9 AND #7  *Indexes=SCI-EXPANDED, SSCI, A&HCI, CPCI-S, CPCI-SSH, ESCI Timespan=All years* | [Edit](https://eur03.safelinks.protection.outlook.com/?url=https%3A%2F%2Fapps.webofknowledge.com%2FWOS_AdvancedSearch_input.do%3Fproduct%3DWOS%26SID%3DD26pnFTNI8xkOpx8KwD%26search_mode%3DAdvancedSearch%26replaceSetId%3D11%26editState%3Dinit&data=01%7C01%7Clisa.azevedo%40kcl.ac.uk%7C49146f88507848e1774c08d81c1d75ef%7C8370cf1416f34c16b83c724071654356%7C0&sdata=vvc0dq7a6LLNntpN%2BpCqOnHolJwqFwhQNaZGZ31F8u0%3D&reserved=0) |  |  |
| --- | --- | --- | --- | --- | --- |
|  | | | | | |
| # 10 | [**72,638**](https://eur03.safelinks.protection.outlook.com/?url=https%3A%2F%2Fapps.webofknowledge.com%2Fsummary.do%3Fproduct%3DWOS%26doc%3D1%26qid%3D12%26SID%3DD26pnFTNI8xkOpx8KwD%26search_mode%3DGeneralSearch%26update_back2search_link_param%3Dyes&data=01%7C01%7Clisa.azevedo%40kcl.ac.uk%7C49146f88507848e1774c08d81c1d75ef%7C8370cf1416f34c16b83c724071654356%7C0&sdata=HMQr%2BHR3oa7ZfDDzS%2FVlwfJJ%2B1jjNk6EfiNzh2XQSHk%3D&reserved=0) | **TOPIC:**  ("virtual real*") *OR* **TOPIC:**  ("vritual-real*") *OR* **TOPIC:**  ("VR") *OR* **TOPIC:**  ("virtual enviro*") *OR* **TOPIC:**  ("virtual character*") *OR* **TOPIC:**  ("VCs") *OR* **TOPIC:**  ("avatar*")  *Indexes=SCI-EXPANDED, SSCI, A&HCI, CPCI-S, CPCI-SSH, ESCI Timespan=All years* | [Edit](https://eur03.safelinks.protection.outlook.com/?url=https%3A%2F%2Fapps.webofknowledge.com%2FWOS_AdvancedSearch_input.do%3Fproduct%3DWOS%26SID%3DD26pnFTNI8xkOpx8KwD%26search_mode%3DAdvancedSearch%26replaceSetId%3D10%26editState%3Dinit&data=01%7C01%7Clisa.azevedo%40kcl.ac.uk%7C49146f88507848e1774c08d81c1d75ef%7C8370cf1416f34c16b83c724071654356%7C0&sdata=JoWi4b7IKLGmfKKIEet1HBuqGvH4VHTjRAcRSlpomec%3D&reserved=0) |  |  |
|  | | | | | |
| # 9 | [**26,347**](https://eur03.safelinks.protection.outlook.com/?url=https%3A%2F%2Fapps.webofknowledge.com%2Fsummary.do%3Fproduct%3DWOS%26doc%3D1%26qid%3D11%26SID%3DD26pnFTNI8xkOpx8KwD%26search_mode%3DGeneralSearch%26update_back2search_link_param%3Dyes&data=01%7C01%7Clisa.azevedo%40kcl.ac.uk%7C49146f88507848e1774c08d81c1d75ef%7C8370cf1416f34c16b83c724071654356%7C0&sdata=2Sbb1iGyi98oiL6T80KB0LQyaOXtgInTh%2F0HY5NEOpo%3D&reserved=0) | **TITLE:**  ("virtual real*") *OR* **TITLE:**  ("vritual-real*") *OR* **TITLE:**  ("VR") *OR* **TITLE:**  ("virtual enviro*") *OR* **TITLE:**  ("virtual character*") *OR* **TITLE:**  ("VCs") *OR* **TITLE:**  ("avatar*")  *Indexes=SCI-EXPANDED, SSCI, A&HCI, CPCI-S, CPCI-SSH, ESCI Timespan=All years* | [Edit](https://eur03.safelinks.protection.outlook.com/?url=https%3A%2F%2Fapps.webofknowledge.com%2FWOS_AdvancedSearch_input.do%3Fproduct%3DWOS%26SID%3DD26pnFTNI8xkOpx8KwD%26search_mode%3DAdvancedSearch%26replaceSetId%3D9%26editState%3Dinit&data=01%7C01%7Clisa.azevedo%40kcl.ac.uk%7C49146f88507848e1774c08d81c1d75ef%7C8370cf1416f34c16b83c724071654356%7C0&sdata=7TdnzDDeleIHZVDBF3J9FnnruIbErvVYj3AyM3n3dcs%3D&reserved=0) |  |  |
|  | | | | | |
| # 8 | [**2,575,623**](https://eur03.safelinks.protection.outlook.com/?url=https%3A%2F%2Fapps.webofknowledge.com%2Fsummary.do%3Fproduct%3DWOS%26doc%3D1%26qid%3D10%26SID%3DD26pnFTNI8xkOpx8KwD%26search_mode%3DGeneralSearch%26update_back2search_link_param%3Dyes&data=01%7C01%7Clisa.azevedo%40kcl.ac.uk%7C49146f88507848e1774c08d81c1d75ef%7C8370cf1416f34c16b83c724071654356%7C0&sdata=e4ZC0jmFyLLWMaIcc5Dt05PbdwTNSfyF5H8CwhlDF18%3D&reserved=0) | **TOPIC:**  ("relax*") *OR* **TOPIC:**  ("autogen*") *OR* **TOPIC:**  ("meditat*") *OR* **TOPIC:**  ("mindful*") *OR* **TOPIC:**  ("rest*") *OR* **TOPIC:**  ("PMR") *OR* **TOPIC:**  ("progressive muscle") *OR* **TOPIC:**  ("breath*") *OR* **TOPIC:**  ("distract*") *OR* **TOPIC:**  ("wellness") *OR* **TOPIC:**  ("wellbeing") *OR* **TOPIC:**  ("well-being")  *Indexes=SCI-EXPANDED, SSCI, A&HCI, CPCI-S, CPCI-SSH, ESCI Timespan=All years* | [Edit](https://eur03.safelinks.protection.outlook.com/?url=https%3A%2F%2Fapps.webofknowledge.com%2FWOS_AdvancedSearch_input.do%3Fproduct%3DWOS%26SID%3DD26pnFTNI8xkOpx8KwD%26search_mode%3DAdvancedSearch%26replaceSetId%3D8%26editState%3Dinit&data=01%7C01%7Clisa.azevedo%40kcl.ac.uk%7C49146f88507848e1774c08d81c1d75ef%7C8370cf1416f34c16b83c724071654356%7C0&sdata=Xr9Jff1Nn4ZKnGM33yE0ovEsuN0I230Y0HEqe%2BfA%2FD8%3D&reserved=0) |  |  |
|  | | | | | |
| # 7 | [**538,004**](https://eur03.safelinks.protection.outlook.com/?url=https%3A%2F%2Fapps.webofknowledge.com%2Fsummary.do%3Fproduct%3DWOS%26doc%3D1%26qid%3D9%26SID%3DD26pnFTNI8xkOpx8KwD%26search_mode%3DGeneralSearch%26update_back2search_link_param%3Dyes&data=01%7C01%7Clisa.azevedo%40kcl.ac.uk%7C49146f88507848e1774c08d81c1d75ef%7C8370cf1416f34c16b83c724071654356%7C0&sdata=TAfA4sOeyvBhb6%2FZip7UhNWmDs8UfulpSBXYVAAvwbI%3D&reserved=0) | **TITLE:**  ("relax*") *OR* **TITLE:**  ("autogen*") *OR* **TITLE:**  ("meditat*") *OR* **TITLE:**  ("mindful*") *OR* **TITLE:**  ("rest*") *OR* **TITLE:**  ("PMR") *OR* **TITLE:**  ("progressive muscle") *OR* **TITLE:**  ("breath*") *OR* **TITLE:**  ("distract*") *OR* **TITLE:**  ("wellness") *OR* **TITLE:**  ("wellbeing") *OR* **TITLE:**  ("well-being")  *Indexes=SCI-EXPANDED, SSCI, A&HCI, CPCI-S, CPCI-SSH, ESCI Timespan=All years* |  |  |  |

*PsychInfo, Embase, Medline (OVID)*

|  |
| --- |
|  |

1. virtual-real*.mp. [mp=title, abstract, original title, name of substance word, subject heading word, floating sub-heading word, keyword heading word, organism supplementary concept word, protocol supplementary concept word, rare disease supplementary concept word, unique identifier, synonyms]
2. VR.mp. [mp=title, abstract, original title, name of substance word, subject heading word, floating sub-heading word, keyword heading word, organism supplementary concept word, protocol supplementary concept word, rare disease supplementary concept word, unique identifier, synonyms]
3. virtual enviro*.mp. [mp=title, abstract, original title, name of substance word, subject heading word, floating sub-heading word, keyword heading word, organism supplementary concept word, protocol supplementary concept word, rare disease supplementary concept word, unique identifier, synonyms]
4. virtual character*.mp. [mp=title, abstract, original title, name of substance word, subject heading word, floating sub-heading word, keyword heading word, organism supplementary concept word, protocol supplementary concept word, rare disease supplementary concept word, unique identifier, synonyms]
5. VCs.mp. [mp=title, abstract, original title, name of substance word, subject heading word, floating sub-heading word, keyword heading word, organism supplementary concept word, protocol supplementary concept word, rare disease supplementary concept word, unique identifier, synonyms]
6. avatar*.mp. [mp=title, abstract, original title, name of substance word, subject heading word, floating sub-heading word, keyword heading word, organism supplementary concept word, protocol supplementary concept word, rare disease supplementary concept word, unique identifier, synonyms]
7. autogen*.mp. [mp=title, abstract, original title, name of substance word, subject heading word, floating sub-heading word, keyword heading word, organism supplementary concept word, protocol supplementary concept word, rare disease supplementary concept word, unique identifier, synonyms]
8. meditat*.mp. [mp=title, abstract, original title, name of substance word, subject heading word, floating sub-heading word, keyword heading word, organism supplementary concept word, protocol supplementary concept word, rare disease supplementary concept word, unique identifier, synonyms]
9. mindful*.mp. [mp=title, abstract, original title, name of substance word, subject heading word, floating sub-heading word, keyword heading word, organism supplementary concept word, protocol supplementary concept word, rare disease supplementary concept word, unique identifier, synonyms]
10. rest*.mp. [mp=title, abstract, original title, name of substance word, subject heading word, floating sub-heading word, keyword heading word, organism supplementary concept word, protocol supplementary concept word, rare disease supplementary concept word, unique identifier, synonyms]
11. PMR.mp. [mp=title, abstract, original title, name of substance word, subject heading word, floating sub-heading word, keyword heading word, organism supplementary concept word, protocol supplementary concept word, rare disease supplementary concept word, unique identifier, synonyms]
12. progressive muscle.mp. [mp=title, abstract, original title, name of substance word, subject heading word, floating sub-heading word, keyword heading word, organism supplementary concept word, protocol supplementary concept word, rare disease supplementary concept word, unique identifier, synonyms]
13. breath*.mp. [mp=title, abstract, original title, name of substance word, subject heading word, floating sub-heading word, keyword heading word, organism supplementary concept word, protocol supplementary concept word, rare disease supplementary concept word, unique identifier, synonyms]
14. distract*.mp. [mp=title, abstract, original title, name of substance word, subject heading word, floating sub-heading word, keyword heading word, organism supplementary concept word, protocol supplementary concept word, rare disease supplementary concept word, unique identifier, synonyms]
15. wellness.mp. [mp=title, abstract, original title, name of substance word, subject heading word, floating sub-heading word, keyword heading word, organism supplementary concept word, protocol supplementary concept word, rare disease supplementary concept word, unique identifier, synonyms]
16. wellbeing.mp. [mp=title, abstract, original title, name of substance word, subject heading word, floating sub-heading word, keyword heading word, organism supplementary concept word, protocol supplementary concept word, rare disease supplementary concept word, unique identifier, synonyms]
17. well-being.mp. [mp=title, abstract, original title, name of substance word, subject heading word, floating sub-heading word, keyword heading word, organism supplementary concept word, protocol supplementary concept word, rare disease supplementary concept word, unique identifier, synonyms]
18. exp Relaxation/
19. virtual real*.mp. [mp=title, abstract, original title, name of substance word, subject heading word, floating sub-heading word, keyword heading word, organism supplementary concept word, protocol supplementary concept word, rare disease supplementary concept word, unique identifier, synonyms]
20. exp Virtual Reality/
21. 1 or 2 or 3 or 4 or 5 or 6 or 19 or 20
22. relax*.mp. [mp=title, abstract, original title, name of substance word, subject heading word, floating sub-heading word, keyword heading word, organism supplementary concept word, protocol supplementary concept word, rare disease supplementary concept word, unique identifier, synonyms]
23. 7 or 8 or 9 or 10 or 11 or 12 or 13 or 14 or 15 or 16 or 17 or 18 or 22
24. 21 and 23
